# Intense ocean freshening from melting glacier around the Antarctica during early twenty-first century

**DOI:** 10.1038/s41598-021-04231-6

**Published:** 2022-01-10

**Authors:** Xianliang L. Pan, Bofeng F. Li, Yutaka W. Watanabe

**Affiliations:** 1 Graduate School of Environmental Science, Hokkaido University, Sapporo, Japan; 2 Faculty of Environmental Earth Science, Hokkaido University, Sapporo, Japan

**Keywords:** Marine chemistry, Cryospheric science

## Abstract

With the accelerating mass loss of Antarctic ice sheets, the freshening of the Southern Ocean coastal oceans (SOc, seas around Antarctica) is gradually intensifying, which will reduce the formation of bottom water and weaken the meridional overturning circulation, thus having a significant negative impact on the ocean’s role in regulating global climate. Due to the extreme environment of the Southern Ocean and the limitations of observational techniques, our understanding of the glacier-derived freshening of SOc is still vague. We developed a method that first provided us with an expansive understanding of glacier-derived freshening progress over the SOc. Applying this method to the observational data in the SOc from 1926 to 2016, revealed that the rate of glacier-derived freshwater input reached a maximum of 268 ± 134 Gt year^−1^ during the early twenty-first century. Our results indicate that during the same period, glacier melting accounted for 63%, 28%, and 92% of the total freshening occurred in the Atlantic, Indian, and Pacific sectors of the SOc, respectively. This suggests that the ice shelf basal melt in West Antarctica and the Antarctic Peninsula plays a dominant role in the freshening of the surrounding seas.

## Introduction

The Antarctic ice sheet accounts for ~ 70% of the freshwater on Earth, equivalent to ~ 60 m of the global sea-level height^1^. With ongoing global warming, the Antarctic ice sheet is losing its mass at a remarkable rate^1,2^. Continuous freshwater input derived from glacier melting would lead to ocean freshening and sea-level rise, which significantly influences the global climate system and long-term climate change^3,4^. In recent years, many studies have demonstrated that the basal melt of the Antarctic ice shelf could explain more than half of the Antarctic ice sheet mass loss^2,5–7^. Because of the emission of anthropogenic materials and the resulting positive trend of the Southern Annular Mode (SAM), the poleward shift of westerly winds and large-scale cyclonic eddies are bringing more and warmer modified Circumpolar Deep Water (mCDW) into Antarctic ice cavities^8–10^, accelerating the basal melt of the Antarctic ice shelf and freshwater export to the ocean^2,6,7,11^. Several studies have found that Antarctic Bottom Water (AABW) has become warmer and fresher, and the formation of AABW has been reduced, which may eventually weaken the meridional overturning circulation of the global ocean^12–16^. Therefore, clarifying the impact of glacier melting on the progress of ocean freshening is important for understanding future climate change.

At present, the correct and expansive estimation of glacier-derived freshening remains bottlenecked due to the severe weather conditions of the Southern Ocean (SO) and limitations of observational techniques. Studies on Antarctic glacier melting and freshening occurring around Antarctica have been primarily based on the following methods. The first method is satellite-based observations^2,4,7,17^. Satellite remote sensing techniques are widely used to monitor Antarctic ice sheet mass balance and sea-level change. The basal melt rate of the Antarctic ice shelf can be directly estimated by subtracting the surface ice discharge from the total mass change of the Antarctic ice sheet. However, this method cannot estimate the effect of glacial melting on ocean freshening. Furthermore, only satellite data from 1979 onward are considered reliable because of the introduction of the multifrequency passive microwave technique^1^. The second method is numerical simulation based on the ocean-sea ice-ice shelf coupled model^9,18^. Numerical simulations can elucidate the physical processes between the ocean and the ice shelf, providing a comprehensive understanding of the current state of Antarctic ice shelf melting based on ice-ocean interactions. However, it is difficult for models to completely reconstruct the complex processes around Antarctica without sufficient physical and chemical parameter observations. The third type of method is in-situ observation. Long-term changes in seawater salinity can describe overall freshening which includes all freshwater sources^12,19–21^. Setting end-members (e.g. δ^18^O and salinity) to characterise each water component is a common way to distinguish different sources of freshwater input^6,18^. However, it is difficult to apply this method on a wide-scale because the end-members of water always change spatiotemporally.

To overcome these limitations and obtain a more comprehensive understanding of Antarctic glacier melting and SOc freshening, parameterization technique has come into our view recently^22–25^. On the one hand, high-accuracy observations of basic hydrographic parameters such as seawater temperature (T), salinity (S), dissolved oxygen (DO), and pressure (Pr) have been conducted for nearly 100 years over the SO with a relatively higher spatiotemporal resolution. Hence, the parameterization constructed by these basic hydrographic parameters enables us to obtain more available data on various chemicals in the SO^24^. On the other hand, if T, S, DO, and Pr are used in the parameterization function, a relationship reflecting the physical (e.g. water mass transportation) and biological (e.g. remineralization) processes can be obtained that relate to the chemical concentration in a specific region. This functional relationship does not change unless it is influenced by any external process. This suggests that for a parameterization of chemical A, the predicted concentration of A (A_pre_) contains a component of the ocean internal processes (A_in_) and a component of the average external process (A_ex_) within the spatiotemporal range of the observed dataset used (Eq. 1). Based on the above two properties, parameterization is often applied to the estimation of external material input in the ocean^24,25^.

1 $${\text{A}}_{{{\text{pre}}}} ({\text{T}},{\text{ S}},{\text{ DO}} \ldots ) \, = {\text{ A}}_{{{\text{in}}}} \left( {{\text{T}},{\text{ S}},{\text{ DO}}} \right) \, + {\text{ A}}_{{{\text{ex}}}} .$$

In this study, we propose a new method based on the interactions between Antarctic glacier and seawater and the oceanic parameterization technique (hereafter referred to as “parameterization method”) that allows us to estimate the glacier-derived freshening without setting any end-member so long as basic ocean hydrographic data (T, S, DO, and Pr) are available. Applying this method to the Southern Ocean coastal oceans (SOc, seas around Antarctica, here defined as the region where the seafloor is shallower than 1500 m, south of 60° S) using ocean hydrographic data set from 1926 to 2016 were collected from the Global Ocean Data Analysis Project version 2 (GLODAPv2) and Southern Ocean Atlas (SOA)^26,27^, we obtained spatial distributions and multi-decadal time series of the glacier-derived freshening over the SOc.

## Results

### Freshwater derived from glacier melting

**Figure 1:**
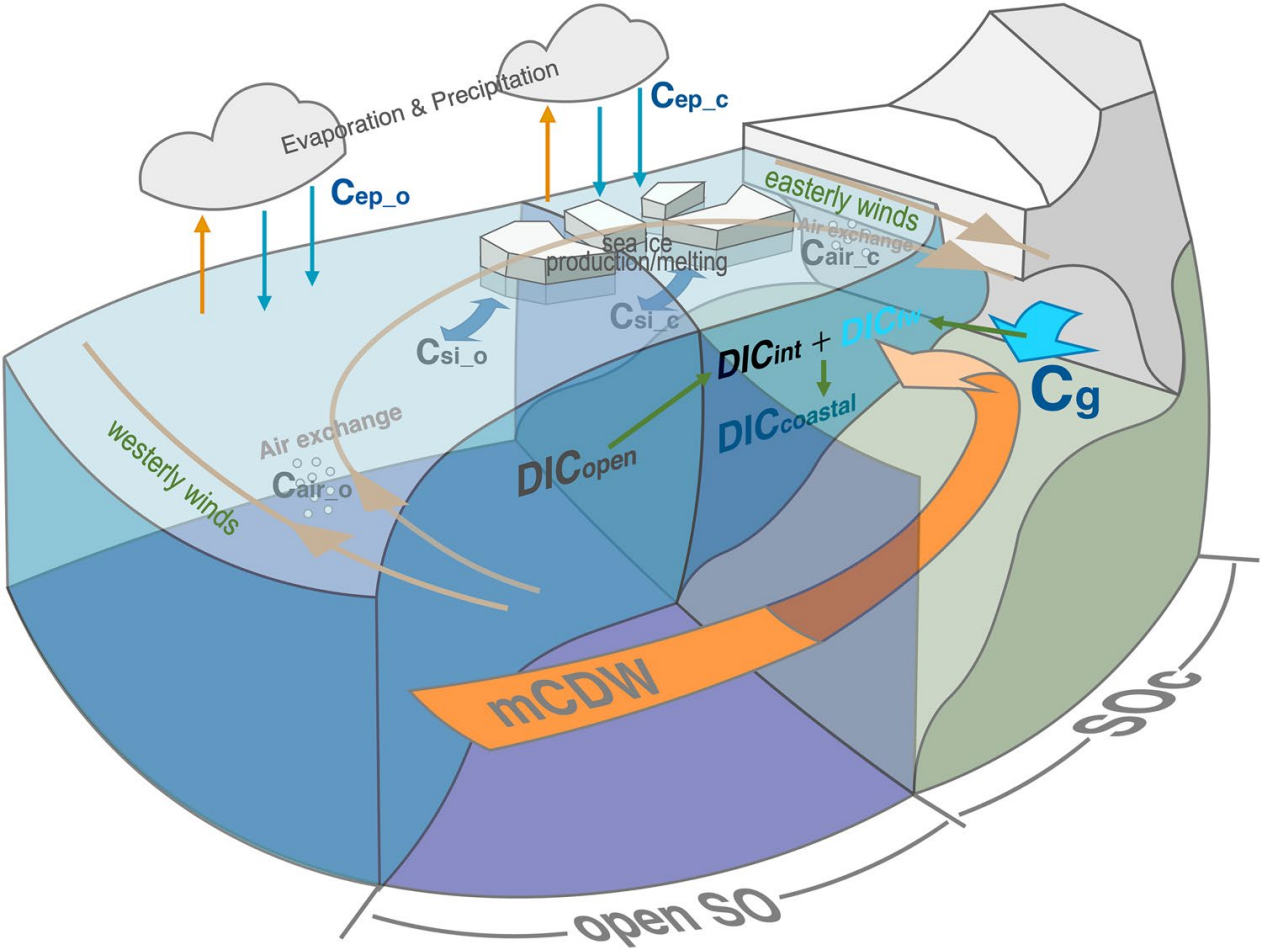
Interactions among the open SO, SOc, and Antarctic glacier. Without any freshwater input from the Antarctic glacier, the initial seawater in the SOc entirely comes from the open SO with a DIC content of DIC_int_. Warm modified CDW (mCDW) inflows from the open SO into the ice cavity southwardly, leading to ice shelf basal melt and freshwater release (with DIC = DIC_fw_; shown by light blue arrows). The buoyant plume of freshwater together with mCDW rises to the surface. The mixture of freshwater and initial seawater makes the DIC concentration in the SOc become DIC_coastal_. ‘C’ indicates the DIC components which is controlled by various external processes; subscripts ‘o’ and ‘c’ indicates processes of the open SO and the SOc, respectively; subscripts ‘ep’, ‘si’, ‘air’ and ‘g’ indicates evaporation and precipitation, sea ice, air-sea exchange, and glacier melting, respectively.

Processes such as glacier melting, sea ice melting, and precipitation release large amounts of freshwater into the SO (Fig. 1). We treated these freshwater input as external process which can change the dissolved inorganic carbon (DIC, used as the indicator of freshening here, see “Methods” for details) concentration in seawater and assumed that this glacier melting is the only significant different external factor between the open ocean of the SO (open SO) and the SOc (see Eqs. 7–21 in the “Methods” section). Then, we constructed the parameterizations of DIC for open SO and SOc, respectively. Both parameterizations can reconstruct DIC using T, S, apparent oxygen utilisation (AOU), and Pr from 0 m to the bottom depth (Eqs. 2 and 3; see Supplementary Fig. S3 and “Methods” for details).

2 $$\begin{aligned}{\mathrm{DIC}}_{\mathrm{open}} & = {a}_{1} +{a}_{2} \cdot {\mathrm{AOU}}_{\mathrm{o}} + {a}_{3} \cdot {\mathrm{T}}_{\mathrm{o}} + {a}_{4} \cdot {\mathrm{S}}_{\mathrm{o}} + {a}_{5} \cdot {\mathrm{Pr}}_{\mathrm{o}} \\ & =1024 + 0.5857 \cdot {\mathrm{ AOU}}_{\mathrm{o}} - 8.452 \cdot {\mathrm{T}}_{\mathrm{o}} + 33.38 \cdot {\mathrm{S}}_{\mathrm{o}} + 1.798\times {10}^{-3} \cdot {\mathrm{Pr}}_{\mathrm{o}}\end{aligned}$$

(Number of data points (n) = 46,753; coefficient of determination (R^2^) = 0.98.

Root-mean-square error (RMSE) = 6.08 µmol kg^−1^)

3 $$\begin{aligned}{\mathrm{DIC}}_{\mathrm{coastal}} & = {b}_{1}+ {b}_{2} \cdot {\mathrm{AOU}}_{\mathrm{c}} + {b}_{3} \cdot {\mathrm{T}}_{\mathrm{c}} + {b}_{4} \cdot {\mathrm{S}}_{\mathrm{c}} + {b}_{5} \cdot {\mathrm{Pr}}_{\mathrm{c}} \\ & = 43.75 + 0.3833 \cdot {\mathrm{AOU}}_{\mathrm{c}} - 4.817 \cdot {\mathrm{T}}_{\mathrm{c}} + 62.43 \cdot {\mathrm{S}}_{\mathrm{c}} \end{aligned}$$

(n = 2059; R^2^ = 0.95; RMSE = 4.84 µmol kg^−1^),

where DIC_open_ indicates the predicted DIC in the open SO; DIC_coastal_ indicates the predicted DIC in the SOc; $a$ and $b$ indicate the regression coefficients for these two parameterizations; subscript ‘o’ indicates parameter of the open SO; subscript ‘c’ represents parameter of the SOc.

Based on the difference in DIC between these two parameterizations, we expressed the fraction of glacier-derived freshwater in the SOc (F_g_), as shown in Eq. (4) (see “Methods” for details).

4 $$\begin{aligned}{\mathrm{F}}_{\mathrm{g}} & = ({\mathrm{DIC}}_{\mathrm{int}} -{\mathrm{DIC}}_{\mathrm{coastal}}) / {\mathrm{DIC}}_{\mathrm{int}} \\ & = [({a}_{1} -{b}_{1}) + ({a}_{2} - {b}_{2}) \cdot {\mathrm{AOU}}_{\mathrm{c}} + ({a}_{3} - {b}_{3}) \cdot {\mathrm{T}}_{\mathrm{c}} + ({a}_{4} -{b}_{4}) \cdot {\mathrm{S}}_{\mathrm{c}} + ({a}_{5} - {b}_{5}) \cdot {\mathrm{Pr}}_{\mathrm{c}}] /({a}_{1} + {a}_{2} \cdot {\mathrm{AOU}}_{\mathrm{c}}+ {a}_{3} \cdot {\mathrm{T}}_{\mathrm{c}} + {a}_{4} \cdot {\mathrm{S}}_{\mathrm{c}} + {a}_{5} \cdot {\mathrm{Pr}}_{\mathrm{c}}),\end{aligned}$$

where DIC_int_ indicates the initial DIC concentration in the SOc without any freshwater input from the Antarctic glacier (Eq. 15). A positive F_g_ indicates freshwater released from the Antarctic glacier to the SOc, leading to freshening. The propagation of error derived from the DIC parameterizations suggests uncertainty in F_g_ of ~ 36%.

### Multi-decadal time-series of seawater freshening over the entire SOc

**Figure 2:**
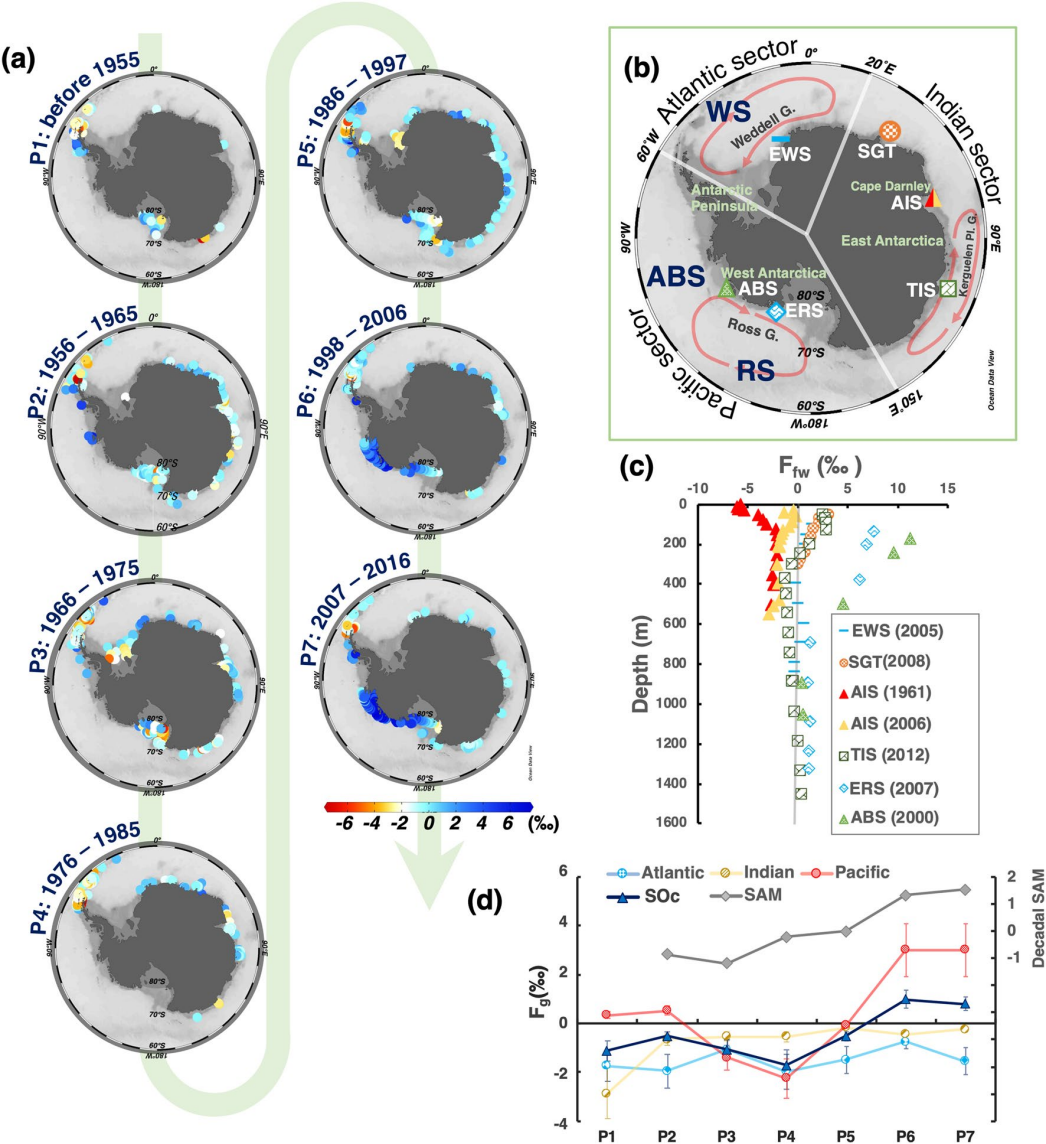
Distributions of glacier-derived freshening over the SOc during 1926–2016. Freshening is represented by the fraction of melt freshwater (F_g_, ‰). Positive values indicate freshwater released from the glacier into the SOc, strengthening the freshening. (**a**) Spatiotemporal distributions of freshening over the SOc. Values are shown as the average F_g_ in the vertical water column. (**b**) Map of the Antarctic and the SO south of 60° S. White lines separate the three sectors of the SOc (Atlantic Sector: 60° W–20° E; Indian Sector: 20° E–150° E; Pacific Sector: 150° E–60° W). Blue abbreviations indicate the following seas: *WS* Weddell Sea, *RS* Ross Sea, *ABS* Amundsen, and Bellingshausen Sea. White abbreviations indicate the stations in Fig. 2c; *EWS* Eastern Weddell Sea, *SGT* Shirase Glacier Tongue, *AIS* Amery Ice Shelf, *TIS* Totten Ice Shelf, *ERS* Eastern Ross Sea, *ABS* Amundsen, and Bellingshausen Seas. (**c**) Vertical distribution of F_g_ in several stations shown in Fig. 2b (white abbreviations with symbols). (**d**) Decadal changes of F_g_ in the three sectors and the entire SOc during 1926–2016 (left axis). Error bars show the uncertainty of 36% derived from the propagation of error. The grey line indicates the decadal change of the Southern Annular Mode (SAM, right axis)^38^. Maps in this figure were drawn using Ocean Data View 5.3.0 (https://odv.awi.de)^47^.

We applied our abovementioned parameterization method, to the observational hydrographic data in the SOc during 1926–2016, which were collected from GLODAPv2 and SOA (23,449 data, almost in summertime)^26,27^, to estimate the time series of freshening (shown as F_g_) over SOc. To obtain the decadal change in freshening, we divided the dataset into seven periods with approximately 10-year intervals (P1,1926–1955; P2,1956–1965; P3,1966–1975; P4, 1976–1985; P5,1986–1997; P6,1998–2006; P7,2007–2016). Regarding spatial division, we divided the SOc into the Atlantic, Indian, and Pacific sectors which are mainly controlled by the ice shelves of the Antarctic Peninsula, East Antarctica, and West Antarctica, respectively (Fig. 2b).

In both the Atlantic and Indian sectors, the averaged F_g_ values were generally near 0 during all periods (Fig. 2a). However, in the Shirase Glacier Tongue (SGT, ~ 38° E) and Totten Ice Shelf (TIS, ~ 116° E), high basal melt rates from the 2000s have been reported^5,6^. Our estimate does show a positive F_g_ in these regions (Fig. 2c). Contrarily, in the Pacific sector, dramatic ice sheet mass loss and high basal melting have been reported to occur in most regions over the last few decades, particularly over the Amundsen and Bellingshausen Seas (ABS, ~ 90° W–150° W)^7,28,29^. Our estimate shows significant positive F_g_ over the Pacific sector, including ABS and Ross Sea, during the late twentieth century to the early twenty-first century (Fig. 2a), which spatiotemporally agrees with the above ice losses. Table 1 shows the rates of glacier-derived freshening (R_g_, Eq. 23) in the three sectors during the seven periods. During P5 to P6, R_g_ in the Pacific sector reached 0.28 ± 0.14‰ year^−1^, while that in the whole SOc was 0.14 ± 0.07‰ year^−1^.

**Table 1:** Rates of glacier-derived freshening (‰ year-^−1^) between each period during 1926 to 2016.

Region	Area (km^2^)	P1–P2^a^	P2–P3	P3–P4	P4–P5	P5–P6	P6–P7
Atlantic	9.8 × 10^5^	− 0.01 ± 0.01^c^	0.09 ± 0.04	− 0.09 ± 0.05	0.05 ± 0.02	0.07 ± 0.03	− 0.08 ± 0.04
Indian	8.6 × 10^5^	0.11 ± 0.06	0.01 ± 0.01	0 ± 0	0.04 ± 0.02	− 0.02 ± 0.01	0.02 ± 0.01
Pacific	1.4 × 10^6^	0.01 ± 0	− 0.19 ± 0.1	− 0.09 ± 0.04	0.22 ± 0.11	0.28 ± 0.14	0 ± 0
SOc^b^	3.3 × 10^6^	0.03 ± 0.01	− 0.05 ± 0.03	− 0.06 ± 0.03	0.12 ± 0.06	0.14 ± 0.07	− 0.02 ± 0.01

^a^P1: 1926–1955, P2: 1956–1965, P3: 1966–1975, P4: 1976–1985, P5: 1986–1997, P6: 1998–2006, P7: 2007–2016.

^b^Rates over the SOc are shown as the area weight average of the three sectors.

^c^Error shows the uncertainty of 50% derived from the RMSE of the parameterizations and the propagation of errors arising in the subsequent calculations.

Figure 2c shows the vertical profiles of F_g_ in several focused regions, which have been reported to have significant ice sheet mass loss. In the Pacific sector, the highest F_g_ was 11 ± 4.0‰ in both ABS and the Eastern Ross Sea (ERS) in the 2000s. In both the Indian and Atlantic sectors, we also found F_g_ reached 4.0 ± 1.5‰ at the surface in the TIS, the SGT, and the Eastern Weddell Sea (EWS). At the Cape Darnley of the Indian sector, which is an important region for sea ice and bottom water formations in East Antarctica^30^, the mass balance of the Amery Ice Shelf (AIS) has recently attracted extensive concern (Fig. 2b)^12,31,32^. In both 1961 and 2006, F_g_ was almost negative [see AIS (1961) and AIS (2006) in Fig. 2c], implying that freshwater exchange is dominated by freshwater consumption due to ice shelf freezing. Comparing the F_g_ in the AIS in 1961 with that in 2006, we identified a positive trend in F_g_ (− 6 ± 2.2‰ to − 1 ± 0.4‰) from 1961 to 2006 (Fig. 2c), implying that ice shelf freezing has been weakened, which may be related to mCDW intrusion^31,32^.

**Figure 3:**
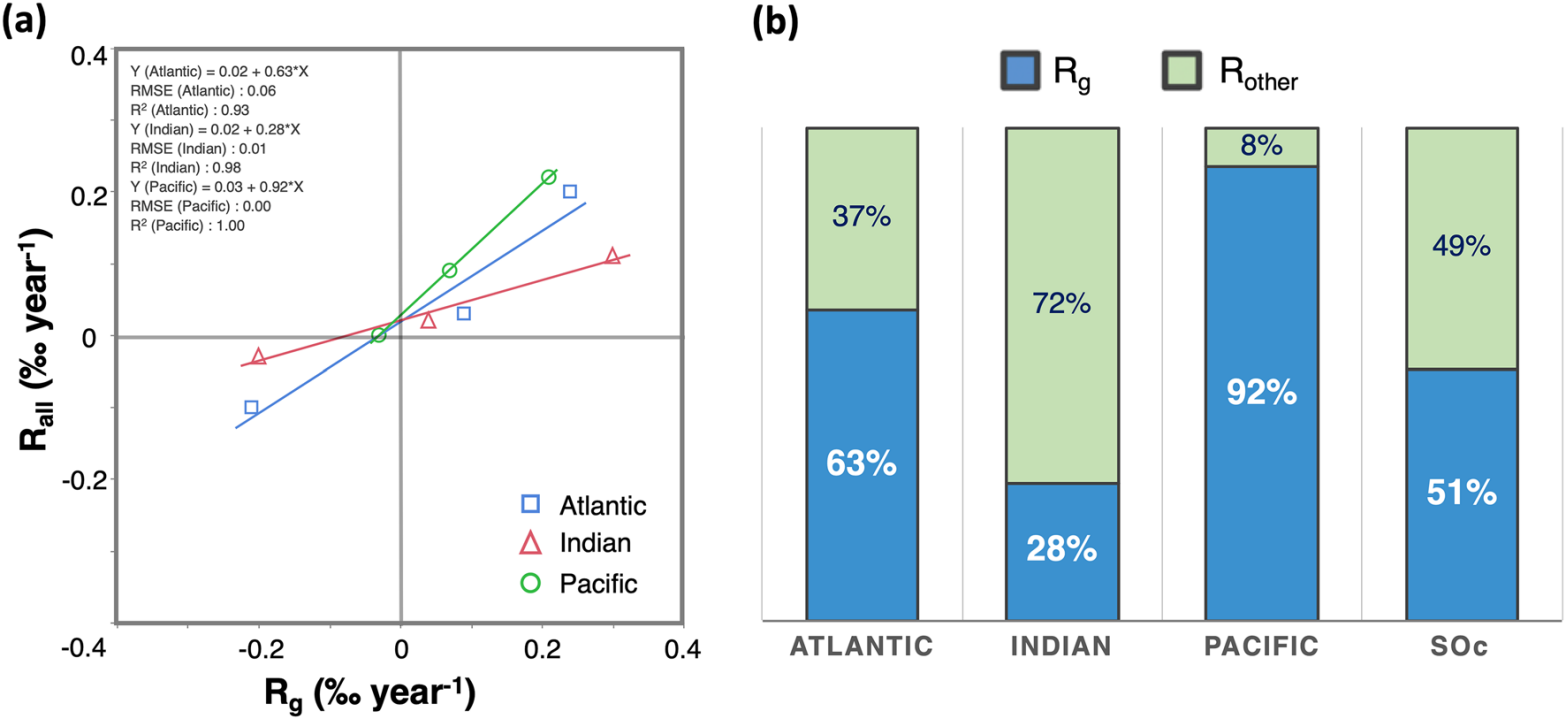
Impact of glacier melting on the SOc. (**a**) Correlations between the rate of glacier-derived freshening (R_g_) and overall freshening (R_all_) in the SOc during 1960 ~ 2016. Blue open squares, red open triangles, and green open circles indicate data picked up from Atlantic, Indian and Pacific sectors of SOc, respectively. Solid lines are the correlation lines of each sector. Data used to plot this figure are given in Supplementary Table S8. (**b**) Proportion of the rate of glacier-derived freshening (R_g_, shown in blue) and freshening derived from other external processes (i.e. evaporation and precipitation and sea ice) (R_other_, shown in green) in each sector and the entire SOc.

To quantify the impact of glacier-derived freshwater on the overall freshening in the SOc, we calculated the rate of overall freshening (R_all_) in the SOc using the salinity trend divided by the average salinity of the research region (Eq. 22). The correlations between R_g_ and R_all_ in the three sectors of the SOc are shown in Fig. 3a. From the slopes of the correlation lines, we found that during 1960–2016, glacier melting accounted for ~ 63%, ~ 28%, and ~ 92% of the total freshening occurred in the Atlantic, Indian, and Pacific sectors of the SOc, respectively (Fig. 3b). This suggests that glacier melting in West Antarctica and the Antarctic Peninsula plays a dominant role in the freshening of the surrounding seas.

We obtained the rate of glacier-derived freshwater input into the SOc by multiplying the regional average R_g_ by the seawater volume. We found that the rate of glacier-derived freshwater input in the SOc reached a maximum of 268 ± 134 Gt year^−1^ (74 ± 37 Gt year^−1^ as the lower limit, Supplementary Text S2) during the late twentieth to early twenty-first centuries (1 Gt = 10^9^ t = 10^12^ kg). If we assume that the melting of ice floe has no significant variation on a decadal time-scale, we can consider that the difference between R_g_ and R_all_ (R_other_ in Fig. 3b) represents the rate of freshwater added by precipitation and melting of newly formed icebergs derived from calving, which have the potential to raise the global sea-level by up to 0.7 ± 0.4 mm year^−1^.

## Discussion

The mass balance of the Antarctic ice sheet is controlled by a combination of several processes^33^. In the Pacific sector, particularly in the ABS, much stronger freshening was observed than in the other two sectors. There is evidence that teleconnections with tropics such as SAM and El Niño–Southern Oscillation (ENSO) contribute significantly to the warm mCDW intrusion and the ice sheet mass loss in the ABS^34^. ABS is located near the eastern limb of the Ross Gyre and is adjacent to the main stem of the Antarctic Circumpolar Current (ACC). This geographic location is very conducive to the intrusion of mCDW into Antarctic ice cavities^28^. The Amundsen Sea Low (low-pressure centre located over the southern Pacific) can also drive the transportation of warm air to West Antarctica, which causes melting of the surface ice sheet and thereby contributes to freshening^35^.

For the Indian and Atlantic sectors, the basal melt rates are generally low because of the typical cold shelf in this region^36^. However, our estimate and several previous studies showed that there were freshening signals in specific regions such as TIS, AIS, and SGT (Fig. 2c)^5,6,31,37^. Except for the effect of the positive SAM, the location and topographical conditions of these areas also play an unneglectable role. For instance, the SGT, which is located at the eastern limb of the Weddell Gyre (Fig. 2b), the deep trough along the continental slope deep into the ice front allows the mCDW to readily touch the ice shelf^6^.

We identified a significant positive correlation between F_g_ over SOc and the SAM index since 1955^38^ (R = 0.82; Fig. 2d and Supplementary Fig. S8), suggesting that a positive SAM is a possible contributor to the Antarctic ice sheet mass loss. Forced by anthropogenic greenhouse gas emissions and stratospheric ozone depletion, SAM has exhibited a positive trend since 1955, resulting in the intensification and southward shift of westerly winds^10,38–40^.

With the development of autonomous ocean observation robotics (Biogeochemical-Argo-float), we can obtain more spatiotemporally complete basic hydrographic data in the SO. Applying our parameterization method to the Bio-Argo-float dataset^41,42^, it will be possible to perform quasi-real-time monitoring of interactions between SOc and Antarctic glaciers and their impacts on the global ocean, which can greatly help us in a deeper understanding of global climate change in the future.

## Methods

### Data used in this study

The observational data used for constructing the parameterizations of DIC (DIC, T, S, DO, and Pr) were sourced from GLODAPv2_2019 of the SO (south of 30° S) from 2000 to 2017^27^. The quality of data on chemicals such as carbon species and nutrients after 2000 was controlled using certified reference materials^43^. Therefore, high-accuracy data for these chemicals began to be obtained mainly after 2000. Basic hydrographic data (T, S, DO, Pr) used to estimate the time series of freshening over the SOc were sourced from GLODAPv2_2019 from 1979 to 2016 and SOA from 1926 to 1984 (south of 60° S, bottom depth shallower than 1500 m)^26,27^. Information on the cruises from which we obtained the data is shown in Supplementary Tables S1, S2, and S3. Quality flags of the World Ocean Circulation Experiment (WOCE) were used to check the quality of the data. In this study, we only used data with a quality flag of two (i.e. the data value is acceptable). To construct the DIC parameterizations, we used 46,753 data points for DIC_open_ and 2059 data points for DIC_coastal_ from 2000 to 2017 (Supplementary Fig. S1). To estimate the time series of freshening, we used 23,449 data points from 1926 to 2016 (Supplementary Fig. S2).

### Construction of DIC parameterizations and its quality validation

We used least-squares multiple linear regression to construct the parameterizations of DIC in the SO, and we established for the first time DIC parameterizations that can be applied to the entire SO from the surface ocean to the seafloor using T, S, AOU, and Pr. AOU was calculated from DO and saturated oxygen concentration^44^. Several constraints were set for the raw data (Supplementary Table S4).

The *F*-test was used to examine the significance of each parameter in our parameterizations (Supplementary Table S5). A parameter with an *F*-value greater than 2.4, was considered to have a significant effect. After a stepwise regression, we selected AOU, T, S, and Pr for the DIC_open_; the *F*-values of each were 375,574, 464,617, 29,712, and 5505, respectively. Conversely, we used AOU, T, and S for the DIC_coastal_, and the *F*-values of each were 4,964,722, and 4080, respectively. The variance inflation factor (VIF) was used to investigate the presence of multicollinearity between each parameter. Standardised regression coefficients (β) were used to compare the contribution of each parameter to DIC (Supplementary Table S5). The closer the absolute value is to 1, the greater the contribution of the parameter. AOU was the most significant parameter in both the open SO and SOc. However, for the significance of T and S, DIC_open_ and DIC_coastal_ show the opposite pattern. The DIC_open_ is mainly controlled by T, while the key parameter becomes S for the DIC_coastal_, partly proving that DIC in the SOc might have been affected by the input of melting freshwater.

We tested the accuracy of our parameterizations by conducting self-validation and cross-validation. First, we used the dataset that was used in the construction of our parameterizations to perform self-validation. Supplementary Figures S6 and S7 show the spatial distributions of the difference between the observed and predicted DIC in the open SO and SOc, respectively. Most circumpolar regions (south of 50° S) showed no significant difference, implying that there are no “blind spots” where our parameterization cannot be applied.

We conducted cross-validation using an independent testing dataset to further verify the reliability of our parameterizations. For DIC parameterization in the open SO (DIC_open_), we selected one independent cruise for each of the three sectors (Atlantic, Indian, and Pacific) that were not used in the construction of parameterization as the testing data set (Supplementary Fig. S4). To quantify the extent of differences in DIC between the independent observed data and DIC_open_, we used the mean absolute deviations (MADs) as follows:

5 $${\mathrm{MAD}}_{\mathrm{open}} = \frac{1}{\mathrm{n}}\sum_{\mathrm{i}=1}^{\mathrm{n}}\left|{\mathrm{DIC}}_{\mathrm{obs}\_\mathrm{i}}-{\mathrm{DIC}}_{\mathrm{open}\_\mathrm{i}}\right|,$$

where MAD_open_ indicates the MAD of DIC_open_, and n is the data amount of each independent testing dataset. MAD_open_ in the Pacific, Indian, and Atlantic sectors were 3.24, 2.48, and 5.06 µmol kg^−1^, respectively (Supplementary Table S6). These MAD values are smaller than the RMSE of DIC_open_ (6.08 µmol kg^−1^), implying that DIC_open_ has sufficient reliability. In contrast, for the parameterization of SOc (DIC_coastal_), the sparseness of observational data makes it difficult to find additional independent testing datasets for accuracy validation. To check the reliability of DIC_coastal_, we used the “*k*-fold cross-validation”^45,46^. The *k*-fold cross-validation uses part of the available data to construct the parameterization (training dataset) and uses the remaining part to test it (testing dataset). Here, we divided the observational data set into 10 roughly equal-sized groups by longitude (i.e. *k* = 10), using one group as the testing dataset and the remaining nine groups as the training data set. We then exchanged other groups as the testing dataset and the remaining nine groups as the training dataset. We repeated the above process nine times. The MAD of DIC_coastal_ (MAD_coastal_) is similar to that in Eq. (5):

6 $${\mathrm{MAD}}_{\mathrm{coastal}} = \frac{1}{\mathrm{n}}\sum_{\mathrm{i}=1}^{\mathrm{n}}\left|{\mathrm{DIC}}_{\mathrm{obs}\_\mathrm{i}}-{\mathrm{DIC}}_{\mathrm{coastal}\_\mathrm{i}}\right|.$$

The results of the *k*-fold cross-validation for the SOc are shown in Supplementary Fig. S5 and Supplementary Table S7.

In the surface layer of both the open SO and the SOc, the differences in DIC between the validation observed data and our parameterizations are relatively large, which is probably due to the air-sea exchange and the seasonal differences between the observational data used.

### Quantification of glacier-derived freshwater input in the SOc

As discussed in the main text, for the parameterization of chemical A, the predicted value of A (A_pre_) contains a term for the ocean internal processes (A_in_) and a term of the average external process (A_ex_) within the spatiotemporal range of the observed dataset used (Eq. 1). Therefore, when we construct parameterizations for DIC in the open SO and SOc, they also satisfy this property (Eqs. 7–9 and 10–12).

7 $${\text{DIC}}_{{{\text{open}}}} = {\text{ DIC}}_{{{\text{in}}\_{\text{o}}}} + {\text{ DIC}}_{{{\text{ex}}\_{\text{o}}}} ,$$

8 $${\text{DIC}}_{{{\text{in}}\_{\text{o}}}} = {\text{ C}}_{{{\text{bio}}\_{\text{o}}}} + {\text{ C}}_{{{\text{phy}}\_{\text{o}}}} ,$$

9 $${\text{DIC}}_{{{\text{ex}}\_{\text{o}}}} = {\text{ C}}_{{{\text{ep}}\_{\text{o}}}} + {\text{ C}}_{{{\text{si}}\_{\text{o}}}} + {\text{ C}}_{{{\text{air}}\_{\text{o}}}} ,$$

10 $${\text{DIC}}_{{{\text{coastal}}}} = {\text{ DIC}}_{{{\text{in}}\_{\text{c}}}} + {\text{ DIC}}_{{{\text{ex}}\_{\text{c}}}} ,$$

11 $${\text{DIC}}_{{{\text{in}}\_{\text{c}}}} = {\text{ C}}_{{{\text{bio}}\_{\text{c}}}} + {\text{ C}}_{{{\text{phy}}\_{\text{c}}}} ,$$

12 $${\text{DIC}}_{{{\text{ex}}\_{\text{c}}}} = {\text{ C}}_{{{\text{ep}}\_{\text{c}}}} + {\text{ C}}_{{{\text{si}}\_{\text{c}}}} + {\text{ C}}_{{{\text{air}}\_{\text{c}}}} + {\text{ C}}_{{\text{g}}} ,$$

where DIC_open_ is defined as the predicted DIC in the open SO; DIC_coastal_ is defined as the predicted DIC in the SOc; subscripts ‘in’ and ‘ex’ indicates terms of DIC concentrations which are controlled by internal processes and external processes of the ocean, respectively; subscripts ‘o and ‘c’ indicates terms of the open SO and the SOc, respectively. DIC_in_ mainly includes two components: the biological components (C_bio_) and the physical components (C_phy_), which can be represented by the parameters (T, S, AOU, Pr). The DIC_ex_ includes the evaporation and precipitation components (C_ep_), sea ice (i.e. floating ice, iceberg) components (C_si_), and air-sea exchange components (C_air_) in both the open SO and the SOc. It is worth noting that in the SOc, there is a unique external DIC component derived from the Antarctic glacier (C_g_).

We quantified the fraction of glacier-derived freshwater in the SOc (F_g_) based on the above parameterizations and processes shown in Fig. 1. The seawater in the SOc consists of two components: one is the seawater coming from the open SO (referred to as initial seawater, with DIC concentration of DIC_int_), and the other is the external freshwater added into the SOc (with DIC concentration of DIC_fw_). The relationship between these water components can be expressed by the following conservation equations:

13 $${\text{F}}_{{{\text{fw}}}} + {\text{ F}}_{{{\text{open}}}} = { 1,}$$

14 $$\begin{aligned} {\text{F}}_{{{\text{fw}}}} \cdot{\text{ DIC}}_{{{\text{fw}}}} + {\text{ F}}_{{{\text{int}}}} \cdot{\text{ DIC}}_{{{\text{int}}}} & = {\text{ F}}_{{{\text{fw}}}} \cdot \, 0 \, + {\text{ F}}_{{{\text{int}}}} \cdot{\text{ DIC}}_{{{\text{int}}}} \\ & = {\text{ DIC}}_{{{\text{coastal}}}} , \\ \end{aligned}$$

where F_int_ is the fraction of the initial seawater. F_fw_ is the fraction of freshwater added to SOc. DIC_fw_ was assumed to be equal to zero.

Assuming that the initial seawater in the SOc comes entirely from the open SO, this allows us to calculate DIC_int_ by substituting the parameters of the SOc into the open ocean parameterization (DIC_open_).

15 $${\text{DIC}}_{{{\text{int}}}} = {\text{ DIC}}_{{{\text{open}}}} \left( {{\text{T}}_{{\text{c}}} ,{\text{ S}}_{{\text{c}}} ,{\text{ AOU}}_{{\text{c}}} ,{\text{ Pr}}_{{\text{c}}} } \right) \, = {\text{ DIC}}_{{{\text{in}}\_{\text{c}}}} + {\text{ DIC}}_{{{\text{ex}}\_{\text{o}}}} .$$

Note that here DIC_in_ is completely controlled by the parameters (T, S, AOU, Pr), so when we substitute the parameters of the SOc into DIC_open_, DIC_in_ becomes DIC_in_c_, whereas DIC_ex_ remains as DIC_ex_o_ because this term is binding to DIC_open_. Combining Eq. (13) with Eq. (14), we obtain F_fw_ as follows.

16 $${\text{F}}_{{{\text{fw}}}} = \, \left( {{\text{DIC}}_{{{\text{int}}}} {-}{\text{ DIC}}_{{{\text{coastal}}}} } \right) \, /{\text{ DIC}}_{{{\text{int}}}} .$$

Then substituting Eqs. (7–9) and (10–12) into Eq. (16), we obtain the following equation:

17 $$\begin{aligned} {\text{F}}_{{{\text{fw}}}} & = \, \left[ {\left( {{\text{DIC}}_{{{\text{in}}\_{\text{c}}}} + {\text{ DIC}}_{{{\text{ex}}\_{\text{o}}}} } \right) \, {-} \, \left( {{\text{DIC}}_{{{\text{in}}\_{\text{c}}}} + {\text{ DIC}}_{{{\text{ex}}\_{\text{c}}}} } \right)} \right] \, /{\text{ DIC}}_{{{\text{int}}}} . \\ & = \, \left[ {\left( {{\text{DIC}}_{{{\text{in}}\_{\text{c}}}} {-}{\text{ DIC}}_{{{\text{in}}\_{\text{c}}}} } \right) \, + \, \left( {{\text{DIC}}_{{{\text{ex}}\_{\text{o}}}} {-}{\text{ DIC}}_{{{\text{ex}}\_{\text{c}}}} } \right)} \right] \, /{\text{ DIC}}_{{{\text{int}}}} . \\ & = \, \left[ {\left( {{\text{C}}_{{{\text{ep}}\_{\text{o}}}} {-}{\text{ C}}_{{{\text{ep}}\_{\text{c}}}} } \right) \, + \, \left( {{\text{C}}_{{{\text{si}}\_{\text{o}}}} {-}{\text{ C}}_{{{\text{si}}\_{\text{c}}}} } \right) \, + \, \left( {{\text{C}}_{{{\text{air}}\_{\text{o}}}} {-}{\text{ C}}_{{{\text{air}}\_{\text{c}}}} } \right) \, {-}{\text{ C}}_{{\text{g}}} } \right] \, /{\text{ DIC}}_{{{\text{int}}}} . \\ \end{aligned}$$

The external components C_ep_, C_si_, and C_air_ exist in both the open SO and SOc. Therefore, we assume that

18 $${\text{C}}_{{{\text{ep}}\_{\text{o}}}} \approx {\text{ C}}_{{{\text{ep}}\_{\text{c}}}} ,$$

19 $${\text{C}}_{{{\text{si}}\_{\text{o}}}} \approx {\text{ C}}_{{{\text{si}}\_{\text{c}}}} ,$$

20 $${\text{C}}_{{{\text{air}}\_{\text{o}}}} \approx {\text{ C}}_{{{\text{air}}\_{\text{c}}}} .$$

Finally, by substituting Eqs. (18–20) into Eq. (17), we obtain F_fw_ as Eq. (21):

21 $${\text{F}}_{{{\text{fw}}}} = \, \left[ {{-}{\text{C}}_{{\text{g}}} } \right] \, /{\text{ DIC}}_{{{\text{int}}}} = {\text{ F}}_{{\text{g}}} .$$

We found that F_fw_ is only controlled by the glacier-derived term, implying that the freshwater estimated by this method can be considered as the freshwater derived from Antarctic glacier melting (F_g_).

We attempted to use various oceanic chemicals, including DIC, nitrate, and phosphate, as indicators of freshwater input. The essential advantage of DIC compared with other chemicals is that it maintains a relatively good linear relationship with hydrographic parameters, even within the surface mixed layer. Especially in the open ocean, the concentration of nutrients in the surface mixed layer is almost zero, which makes it difficult to construct parameterizations. Therefore, DIC was chosen as the freshwater indicator.

The average F_g_ in each sector of the SOc shown in Fig. 2d were calculated after gridding the raw data shown in Fig. 2a. This is done to lower the impact of spatial bias in the raw data distribution. We interpolated the raw data onto a 1° × 1° grid with a scale-length of 5° of longitude and 1° of latitude using the Ocean Data View software (Supplementary Fig. S10)^47^. The area of each grid was also considered when calculating the average, since the area of the grid varies with latitude. The average F_g_ of the SOc is shown as the area weight average of the three sectors.

We changed the fraction of freshwater into volume by multiplying the fraction by the seawater volume. The seawater volumes used here were calculated by multiplying the average depth of all data profiles by the ocean surface area of the SOc or the three sectors. The ocean surface areas are listed in Table 1.

### Calculation of the rate of overall freshening in the SOc based on the salinity trend

To quantify the impact of glacier melting on the SOc, we calculated the rate of overall freshening in the SOc according to the following steps: It is impossible to estimate the fraction of freshwater at a given moment through the salinity. Thus, we simply estimated the rate of freshening based on the rate of salinity change.

22 $${\text{R}}_{{{\text{all}}}} \, = \,\left( {{\text{dS}}_{{{\text{obs}}}} /{\text{dt}}} \right) \, /{\text{ S}}_{{{\text{ave}}}} ,$$

where R_all_ indicates the rate of overall freshening in the SOc; dS_obs_/dt indicates the observed salinity trend, which is controlled by evaporation and precipitation, sea ice, and glaciers, and S_ave_ is the average salinity over our research region (S_ave_ = 34.3).

The rate of glacier-derived freshening (R_g_) is calculated as follow:

23 $${\text{R}}_{{\text{g}}} = {\text{ dF}}_{{\text{g}}} /{\text{dt}}{.}$$

## Supplementary Information

Supplementary Information.

## Data Availability

Hydrographic data and DIC data after 2000 used to construct DIC parameterizations are available in GLODAP v2 2020 (https://www.glodap.info/index.php/data-access/). Hydrographic data from 1926 to 2016 used to estimate freshwater input are available in GLODAP v2 2020 and Southern Ocean Atlas (https://odv.awi.de/data/ocean/southern-ocean-atlas/). See a detailed description of the data used in this study in the Methods section.
