# Supplementary material for: Intense ocean freshening from melting glacier around the Antarctica during early twenty-first century
Source: Sci Rep. 2022 Jan 10;12:383. doi: 10.1038/s41598-021-04231-6 (PMC8748732; doi:10.1038/s41598-021-04231-6)
Supplement: Supplementary file 1 — Supplementary Information. [file 41598_2021_4231_MOESM1_ESM.docx]

**Intense ocean freshening from melting glacier around the Antarctica during early 21^st^ century**

Xianliang L. Pan^1*^, Bofeng F. Li^2^, Yutaka W. Watanabe^2^

^1^ Graduate School of Environmental Science, Hokkaido University, Sapporo, Japan

^2^ Faculty of Environmental Earth Science, Hokkaido University, Sapporo, Japan

* Corresponding author: [panxianliang@ees.hokudai.ac.jp](mailto:panxianliang@ees.hokudai.ac.jp)

Supplementary information

Text S1. Interactions among the open SO, SOc, and Antarctic ice sheet

Figure 1 shows the interactions among the open SO, SOc, and Antarctic glaciers. Initially, without any freshwater input from the Antarctic glacier,　the seawater in SOc is supplied by open SO, implying that the processes which control the DIC concentration in seawater are all included in the DIC parameterization of open SO (DIC_open_). Therefore, the DIC in the SOc can be expressed by substituting the parameters of SOc into the DIC parameterization of the open SOc. The warm mCDW (~1 °C) inflows from the open SO into the ice cavity southward. The freezing point of seawater at a depth of 1000 m was at ca. –3 ˚C. The difference in temperature of approximately 4 °C between the mCDW and the freezing point near the deep ice shelves has the potential to cause rapid ice shelf basal melt and freshwater release. The buoyant plume of freshwater, together with the mCDW, rose upward. The mixture of freshwater and seawater flowed northward along the surface ice shelf. The mixture of freshwater and initial seawater causes the DIC concentration in the SOc to become DIC_coastal_. Other external processes such as sea ice production and melting, evaporation and precipitation, and air exchange can also change the DIC content in seawater, but these processes occur in both open SO and SOc, so they will not cause significant differences between DIC_open_ and DIC_coastal_.

We assumed DIC_open_ to be negligibly affected by the glacier melting effect, because it is necessary to consider a 1000-year time scale, at the least, for the coastal water to completely spread to the open ocean ^1^.

Text S2. Lack of observational data in ABS before P6

When we estimated the rate of glacier-derived freshening (R_g_) in the three sectors of SOc, we divided the observational dataset into seven periods with approximately 10-year intervals (P1:1926–1955; P2:1956–1965; P3:1966–1975; P4: 1976–1985; P5:1986–1997; P6:1998–2006; P7:2007–2016), and calculated the R_g_ between every two adjacent periods. We noted that data of ABS before P6 is quite limited, which could bring uncertainty to our estimate in the Pacific sector if only taking the difference between the adjacent two periods (e.g. Fig. 2d, P5 to P6). Taking this uncertainty into account, we provided a possible range of R_g_ for the Pacific sector, with a lower limit of the average R_g_ during P2 to P6, since ABS data do exist in these two periods. For example, from P5 to P6, R_g_ reached a maximum of 268 ± 134 Gt year^-1^ (calculated by the change in F_g_ from P5 to P6), with a lower limit of 74 ± 37 Gt year^-1^ (calculated by the average change in F_g_ from P2 to P6).

Text S3. Rationality of assumptions in this study

In this section, we discuss the rationality of the main assumptions made in this study: (i) Glacier melting is the only significant external possess which can influence the DIC content in the seawater between SOc and the open ocean of SO. (ii) All freshwater derived from the Antarctic ice sheet can be included in the SOc (bottom depth is shallower than 1,500 m, south of 60 ° S). (iii) The absorption of anthropogenic DIC (DIC_ant_) had no significant impact on our estimate of freshening; (iv) DIC concentration was negligible in the melt-water from the surface ice sheet. We explain the rationality of these assumptions in detail as follows:

(i) This is the fundamental assumption of this study. We derived this in detail in the Methods section (equation S3 to equation S11) why the results obtained by our method represent the fraction of glacial meltwater. It is worth noting that the characteristics of the Southern Ocean vary significantly in the meridional direction, as many of the parameters of SO are distributed in a ribbon pattern along several fronts (e.g. the polar front, between approximately 50° and 60°S). For instance, sea ice coverage and air-sea exchange are obviously different between the north and south of the polar front. This seems to challenge our assumptions. In this study, SOc is defined as the region south of 60 °S and bottom depth shallower than 1500 m. Except for the Drake Passage, the boundary between SOc and open SO is further south than the polar front (~65 °S to 70 °S). Thus, the parameterization of open SO can reflect most of the processes controlling DIC concentration in seawater south of the polar front (except for glacier melting).

(ii) We wanted to estimate, as accurately as possible, the discharge of all freshwater derived from the Antarctic ice sheet into SOc. Therefore, for the definition of the SOc domain, we chose a bottom depth of 1,500 m as the boundary. Fig. S9 shows four north-south vertical sections of the freshwater fraction (F_g_) estimated in this study around the SOc. We can see that the F_g_ at the surface gradually decreases with increasing distance from the Antarctic ice sheet and decreases to nearly zero before reaching the boundary of SOc. We can therefore assume that all significant freshwater inputs were confined to the SOc defined in this study.

(iii) Regarding the influence of DIC_ant_ on the DIC concentration in the SO, recent studies have reported that anthropogenic DIC in the SO increased at an average rate of ~1 μmol kg^-1^ year^-1^ from 1994 to 2007 ^2^. Considering the RMSE of our parameterizations, the propagation of error, and the time scale, our estimate can only be influenced by processes which lead to DIC change > 1 μmol kg^-1^ year^-1^. Furthermore, there are anthropogenic terms in both the parameterization of SOc and open SO. The effect of DIC_ant_ can be cancelled out to some extent when calculating the fraction of freshwater (equation S9). Therefore, even if there is an effect of DIC_ant_, this is not significant in our results.

(iv) The DIC concentrations in the meltwater from the surface ice sheet were assumed to be zero. The meltwater derived from the surface Antarctic ice sheet is in contact with the atmosphere and absorbs atmospheric CO_2_ before flowing to the SOc. Consequently, the DIC concentration in the meltwater was not zero. If the meltwater has S = 0 and T = 0 in complete equilibrium with an atmospheric CO_2_ of 410 ppm at present, the meltwater will have a DIC of 36 μmol kg^-1^, which is approximately 1.6% of the average level of DIC in seawater. The actual concentration of DIC in the meltwater must be lower than this value because it takes a long time to become saturated with atmospheric CO_2_ ^3^. Therefore, it is reasonable to neglect the DIC in surface ice sheet meltwaters.

Tables

**Table S1.** Information of cruises undertaken for constructing DICopen from GLODAPv2_2019 during 2000–2017 in this study ^4^. The cruise data map is shown in Fig. S1.

| **EXPOCODE** | **Date** | **Sector** | **Data amount** | **EXPOCODE** | **Date** | **Sector** | **Data amount** |
| --- | --- | --- | --- | --- | --- | --- | --- |
| 06AQ20021124 | 2002-11 | Atlantic | 1619 | 35MF20000117 | 2000-01 | Indian | 172 |
| 06AQ20050122 | 2005-01 | Atlantic | 2012 | 35MF20000719 | 2000-07 | Indian | 66 |
| 06AQ20060825 | 2006-08 | Atlantic | 289 | 35MF20010103 | 2001-01 | Indian | 108 |
| 06AQ20071128 | 2007-11 | Atlantic | 776 | 35MF20020104 | 2002-01 | Indian | 149 |
| 06AQ20080210 | 2008-02 | Atlantic | 1800 | 35MF20040103 | 2004-01 | Indian | 106 |
| 06AQ20101128 | 2010-11 | Atlantic | 1618 | 35MF20050111 | 2005-01 | Indian | 79 |
| 06AQ20120107 | 2012-01 | Atlantic | 674 | 35MF20090103 | 2009-01 | Indian | 73 |
| 06AQ20141202 | 2014-12 | Atlantic | 619 | 35MF20091219 | 2009-12 | Indian | 144 |
| 06M220170104 | 2017-01 | Atlantic | 812 | 35MF20110114 | 2011-01 | Indian | 93 |
| 33RO20050111 | 2005-01 | Atlantic | 860 | 49NZ20130106 | 2013-01 | Indian | 1063 |
| 33RO20100308 | 2010-03 | Atlantic | 817 | 61TG20020206 | 2002-02 | Indian | 85 |
| 33RO20110926 | 2011-09 | Atlantic | 670 | 61TG20030217 | 2003-02 | Indian | 48 |
| 33RO20131223 | 2013-12 | Atlantic | 923 | 74AB20020301 | 2002-03 | Indian | 38 |
| 35MF20080207 | 2008-02 | Atlantic | 1051 | 74DI20041103 | 2004-11 | Indian | 217 |
| 49NZ20031106 | 2003-11 | Atlantic | 496 | 74DI20041213 | 2004-12 | Indian | 68 |
| 740H20081226 | 2008-12 | Atlantic | 470 | 096U20160426 | 2016-04 | Pacific | 1059 |
| 740H20090307 | 2009-03 | Atlantic | 50 | 09FA20010524 | 2001-05 | Pacific | 268 |
| 74JC20100319 | 2010-03 | Atlantic | 479 | 09SS20090203 | 2009-02 | Pacific | 278 |
| 74JC20151217 | 2015-12 | Atlantic | 72 | 316N20050821 | 2005-08 | Pacific | 2084 |
| 096U20150321 | 2015-03 | Indian | 53 | 318M20091121 | 2009-11 | Pacific | 1667 |
| 096U20160108 | 2016-01 | Indian | 38 | 33LG20060321 | 2006-03 | Pacific | 154 |
| 096U20160314 | 2016-03 | Indian | 35 | 33RO20071215 | 2007-12 | Pacific | 1279 |
| 09AR20011029 | 2001-10 | Indian | 1074 | 33RO20161119 | 2016-11 | Pacific | 1510 |
| 09AR20041223 | 2004-12 | Indian | 806 | 33RR20050109 | 2005-01 | Pacific | 1607 |
| 09AR20060102 | 2006-01 | Indian | 885 | 49HH20011127 | 2001-11 | Pacific | 108 |
| 09AR20071216 | 2007-12 | Indian | 487 | 49NZ20030803 | 2003-08 | Pacific | 1228 |
| 09AR20080322 | 2008-03 | Indian | 979 | 49NZ20071122 | 2007-11 | Pacific | 36 |
| 09FA20000926 | 2000-09 | Indian | 223 | 49NZ20121128 | 2012-11 | Pacific | 952 |
| 33RR20070204 | 2007-02 | Indian | 1698 | 49NZ20170208 | 2017-02 | Pacific | 329 |
| 33RR20080204 | 2008-02 | Indian | 1124 | 740H20090203 | 2009-02 | Pacific | 760 |
| 33RR20090320 | 2009-03 | Indian | 2394 | 32O620110219 | 2011-02 | Pacific | 2310 |
| 33RR20160208 | 2016-02 | Indian | 1324 | 32O620140320 | 2014-03 | Pacific | 1388 |

**Table S2.** Information of cruises undertaken for constructing DIC_coastal_ from GLODAPv2_2019 during 2000–2017 in this study ^4^. The cruise data map is shown in Fig. S1.

| **EXPOCODE** | **Date** | **Sector** | **Data amount** |
| --- | --- | --- | --- |
| 06AQ20050122 | 2005-01 | Atlantic | 160 |
| 06AQ20060825 | 2006-08 | Atlantic | 165 |
| 06AQ20080210 | 2008-02 | Atlantic | 78 |
| 06AQ20101128 | 2010-11 | Atlantic | 70 |
| 32O620110219 | 2011-02 | Atlantic | 130 |
| 74JC20100319 | 2010-03 | Atlantic | 121 |
| 06AQ20021124 | 2002-11 | Indian | 9 |
| 09AR20011029 | 2001-10 | Indian | 131 |
| 09AR20041223 | 2004-12 | Indian | 57 |
| 09AR20060102 | 2006-01 | Indian | 208 |
| 09AR20071216 | 2007-12 | Indian | 647 |
| 09AR20080322 | 2008-03 | Indian | 24 |
| 09AR20160111 | 2016-01 | Indian | 1 |
| 33RR20070204 | 2007-02 | Indian | 29 |
| 33RR20080204 | 2008-02 | Indian | 125 |
| 33RR20160208 | 2016-02 | Indian | 31 |
| 49NZ20130106 | 2013-01 | Indian | 23 |
| 61TG20020206 | 2002-02 | Indian | 16 |
| 61TG20030217 | 2003-02 | Indian | 16 |
| 740H20090203 | 2009-02 | Pacific | 18 |

| **Table S3.** Information of cruises undertaken for estimating the time-series of freshening over the SOc from GLODAPv2_2019 and SOA during 1926–2016 in this study ^4,5^. The cruise data map is shown in Fig. S2. | | | | | | | |
| --- | --- | --- | --- | --- | --- | --- | --- |
| **EXPOCODE** | **Date** | **Sector** | **Data amount** | **EXPOCODE** | **Date** | **Sector** | **Data amount** |
| Argentina_Cap. Canep | 1963 to 1965 | Atlantic | 68 | USSR_Langust | 1967-01 | Atlantic | 23 |
| Argentina_Gen. San-M | 1954 to 1966 | Atlantic | 149 | USSR_Muksun | 1964-02 | Atlantic | 27 |
|  | 1969-02 |  |  | USSR_Orehovo | 1965-02 | Atlantic | 54 |
|  | 1971-01 |  |  | USSR_Pioner Latvii | 1989-02 | Atlantic | 300 |
| Argentina_Islas Orca | 1975 to 1976 | Atlantic | 235 | USSR_Prof. Vieze | 1971-02 | Atlantic | 292 |
|  | 1978-05 |  |  |  | 1986-02 |  |  |
| FRG_Meteor | 1926-01 | Atlantic | 10 | USSR_Prof. Zubov | 1971-01 | Atlantic | 145 |
| Germany_Jan Wellem | 1937-03 | Atlantic | 25 |  | 1974-12 |  |  |
| Japan_Fuji-Maru | 1968 to 1969 | Atlantic | 222 |  | 1977-02 |  |  |
|  | 1973-03 |  |  |  | 1978-01 |  |  |
|  | 1980-01 |  |  | Japan_Shirase | 1984 to 1988 | Indian | 324 |
| U.K._Discovery II | 1930 to 1933 | Atlantic | 723 | Japan_Umitaka-Maru | 1964-12 | Indian | 324 |
|  | 1936-01 |  |  |  | 1966-12 |  |  |
|  | 1936-11 |  |  |  | 1970-01 |  |  |
|  | 1938-03 |  |  |  | 1977-12 |  |  |
|  | 1939-03 |  |  |  | 1984-01 |  |  |
| USA_Burton Island | 1958-01 | Atlantic | 348 | Norway_Thorshaven | 1934-01 | Indian | 11 |
|  | 1962-01 |  |  | U.K._Discovery | 1931-01 | Indian | 7 |
| USA_Glacier | 1957-11 | Atlantic | 591 | unknown_unknown | 1973-02 | Indian | 200 |
|  | 1960-03 |  |  |  | 1974-12 |  |  |
|  | 1962-02 |  |  |  | 1977-03 |  |  |
|  | 1968 to 1970 |  |  |  | 1978-01 |  |  |
|  | 1973 to 1976 |  |  | USA_Eltanin | 1963-05 | Indian | 999 |
| USA_Knorr | 1984-02 | Atlantic | 33 |  | 1964-03 |  |  |
| USA_Melville | 1975-02 | Atlantic | 333 |  | 1967 to 1972 |  |  |
|  | 1981-01 |  |  | USSR_Acad. Feodorov | 1988-01 | Indian | 244 |
| USA_R.D. Conrad | 1974-01 | Atlantic | 59 |  | 1989-01 |  |  |
|  | 1975-02 |  |  | USSR_Chatyr-Dag | 1978-02 | Indian | 94 |
| USA_Thomas Washingto | 1969-01 | Atlantic | 173 | USSR_Mihail Somov | 1978-04 | Indian | 250 |
|  | 1971-02 |  |  |  | 1981-11 |  |  |
| USSR_Gizhiga | 1972-12 | Atlantic | 867 | USA_T.G.Thompson | 1976-03 | Pacific | 107 |
|  | 1978-12 |  |  | USA_unknown | 1961-01 | Pacific | 30 |

| **Table S3.** Continued. | | | | | | | |
| --- | --- | --- | --- | --- | --- | --- | --- |
| **EXPOCODE** | **Date** | **Sector** | **Data amount** | **EXPOCODE** | **Date** | **Sector** | **Data amount** |
| USSR_Acad. Knipovich | 1967-02 | Atlantic | 391 | USSR_Ob | 1956 to 1963 | Indian | 1627 |
|  | 1969 to 1970 |  |  |  | 1965 to 1968 |  |  |
|  | 1974-12 |  |  | USSR_Salehard | 1980-02 | Indian | 52 |
|  | 1978-12 |  |  | Chile_Yelcho | 1964-02 | Pacific | 87 |
| USSR_Evrica | 1981-02 | Atlantic | 74 | Norway_Brategg | 1947-12 | Pacific | 96 |
| 06AQ19890906 | 1989-09 | Atlantic | 143 | USA_Atka | 1964-02 | Pacific | 190 |
| 06AQ19901117 | 1990-11 | Atlantic | 70 | 33RR20160208 | 2016-02 | Indian | 61 |
| 06AQ19920521 | 1992-05 | Atlantic | 162 | 35MF19850224 | 1985-02 | Indian | 20 |
| 06AQ19921203 | 1992-12 | Atlantic | 199 | 35MF19930123 | 1993-01 | Indian | 35 |
| 06AQ19960317 | 1996-03 | Atlantic | 61 | 35MF19960220 | 1996-02 | Indian | 29 |
| 06AQ19980328 | 1998-03 | Atlantic | 184 | 49HH19941213 | 1994-12 | Indian | 14 |
| 06AQ20050122 | 2005-01 | Atlantic | 213 | 49NZ20130106 | 2013-01 | Indian | 23 |
| 06AQ20060825 | 2006-08 | Atlantic | 244 | 49ZS19921203 | 1992-12 | Indian | 24 |
| 06AQ20080210 | 2008-02 | Atlantic | 242 | 61TG20020206 | 2002-02 | Indian | 16 |
| 06AQ20101128 | 2010-11 | Atlantic | 70 | 61TG20030217 | 2003-02 | Indian | 29 |
| 06AQ20141202 | 2014-12 | Atlantic | 327 | 90KD19920214 | 1992-02 | Indian | 78 |
| 316N19831007 | 1983-10 | Atlantic | 36 | 32O619960503 | 1996-05 | Indian | 109 |
| 32L919940204 | 1994-02 | Atlantic | 222 | 06MT19900123 | 1990-01 | Pacific | 8 |
| 58A119890214 | 1989-02 | Atlantic | 87 | 29HE19951203 | 1995-12 | Pacific | 346 |
| 74JC19950320 | 1995-03 | Atlantic | 17 | 29HE19960117 | 1996-01 | Pacific | 115 |
| 74JC19990315 | 1999-03 | Atlantic | 253 | 33RR19971020 | 1997-10 | Pacific | 15 |
| 74JC20100319 | 2010-03 | Atlantic | 585 | 33RR19971202 | 1997-12 | Pacific | 35 |
| 74JC20151217 | 2015-12 | Atlantic | 33 | 740H20090203 | 2009-02 | Pacific | 38 |
| 74JC20161110 | 2016-11 | Atlantic | 18 | 32O619970113 | 1997-01 | Pacific | 1257 |
| 06AQ20021124 | 2002-11 | Indian | 11 | 32O619970404 | 1997-04 | Pacific | 552 |
| 09AR19930404 | 1993-04 | Indian | 24 | 32O620000215 | 2000-02 | Pacific | 934 |
| 09AR19940101 | 1994-01 | Indian | 178 | 32O620070203 | 2007-02 | Pacific | 964 |
| 09AR19941213 | 1994-12 | Indian | 125 | 32O620110219 | 2011-02 | Pacific | 204 |
| 09AR19960119 | 1996-01 | Indian | 434 | 09AR20080322 | 2008-03 | Indian | 55 |
| 09AR20011029 | 2001-10 | Indian | 329 | 09AR20110104 | 2011-01 | Indian | 1546 |
| 09AR20030103 | 2003-01 | Indian | 157 | 09AR20120105 | 2012-01 | Indian | 190 |
| 09AR20041223 | 2004-12 | Indian | 110 | 09AR20160111 | 2016-01 | Indian | 26 |
| 09AR20060102 | 2006-01 | Indian | 393 | 33RR20070204 | 2007-02 | Indian | 29 |
| 09AR20071216 | 2007-12 | Indian | 1778 | 33RR20080204 | 2008-02 | Indian | 210 |

| **Table S4.** Constraint conditions for DIC parameterizations. | | | |
| --- | --- | --- | --- |
| **Region** | **Constraints** |  | **Remarks** |
| open SO | Bottom depth | > 1500 m |  |
|  | AOU | Remove  deeper than 1000m &  AOU< 165 μmol kg^-1^ |  |
|  | Salinity | 34 – 35 |  |
|  | Water masses | Remove  NADW ^a^, | NADW: 34.8 < S < 35 |
|  |  |  | 1.5 ℃ < T < 4 ℃ |
|  |  | SASW ^b^ | SASW: T > 8 ℃ ^6^ |
| coastal SO (SOc) | Bottom depth | ≤ 1500 m |  |
|  | Salinity | 34 – 35 |  |

**^a^** North Atlantic Deep Water

**^b^** Sub-Antarctic Surface Water. In this study, we only removed SASW in the Pacific sector.

| **Table S5.** Summary of parameterizations of DIC in this study. | | | | | |
| --- | --- | --- | --- | --- | --- |
| **Region** | **Parameter** | ***F* ^a^** | ***B* ^b^** | **Standardized *β* ^c^** | **VIF ^d^** |
| open SO  (DIC_open_) | Intercept | – | 1024 | – | – |
|  | AOU | 375,574 | 0.5857 | 0.53 | 2.09 |
|  | T | 464,617 | – 8.452 | – 0.5 | 1.54 |
|  | S | 29,712 | 33.38 | 0.15 | 2 |
|  | Pr | 5,505 | 1.798×10^-3^ | 0.05 | 1.51 |
| coastal SO (DIC_coastal_) | Intercept | – | 43.75 | – | – |
|  | AOU | 4,964 | 0.3833 | 0.64 | 2.64 |
|  | T | 722 | – 4.817 | – 0.19 | 1.54 |
|  | S | 4,080 | 62.43 | 0.5 | 1.95 |

**^a^** *F*-value with a significance level of α = 0.05; significant when *F*-value over 2.4.

**^b^** Regression coefficient

**^c^** Standardized regression coefficient

**^d^** Variance Inflation Factor; indicates no multicollinearity when VIF is below 10

| **Table S6.** Mean Absolute Deviation (MAD) of difference between DIC_obs_ and DIC_open_ in three independent cruises along the Atlantic, Indian, and Pacific sectors in the open SO. | | | |
| --- | --- | --- | --- |
| **Region** | **Cruise** | **n ^a^** | **MAD_open_ (μmol kg^-1^)** |
| Atlantic Sector | 06AQ20071128 | 111 | 5.06 |
| Indian Sector | 09AR20160111 | 97 | 2.48 |
| Pacific Sector | 49NZ20170208 | 141 | 3.24 |
| RMSE of DIC_open_ (μmol kg^-1^) | 6.08 | | |

**^a^** Number of data points

| **Table S7.** Result of the *k*-fold cross-validation of DIC_coastal_. | | | |  |  |  |
| --- | --- | --- | --- | --- | --- | --- |
| **Testing set** | **n_test ^a^** | **Longitude** | **Parameterization** | **n_train ^b^** | **RMSE (μmol kg^-1^)** | **MADcoastal (μmol kg^-1^)** |
| 1 | 200 | 178˚W – 55˚W | DIC = –11.25 – 5.149×T + 64.02×S + 0.3850×AOU | 1859 | 4.75 | 4.23 |
| 2 | 200 | 55˚W – 53˚W | DIC = 29.06 – 5.050×T + 62.85×S + 0.3836×AOU | 1859 | 4.74 | 3.91 |
| 3 | 200 | 53˚W – 44˚W | DIC = –7.260 – 5.028×T + 63.91×S + 0.3792×AOU | 1859 | 4.57 | 4.92 |
| 4 | 200 | 44˚W – 31˚E | DIC = 49.32 – 4.786×T + 62.29×S + 0.3762×AOU | 1859 | 4.76 | 3.74 |
| 5 | 200 | 31˚E – 70˚E | DIC = 34.09 – 4.893×T + 62.71×S + 0.3859×AOU | 1859 | 4.98 | 2.59 |
| 6 | 200 | 70˚E – 139˚E | DIC = 59.60 – 4.654×T + 61.97×S + 0.3868×AOU | 1859 | 4.80 | 3.95 |
| 7 | 200 | 139˚E – 140˚E | DIC = 79.95 – 4.828×T + 61.38×S + 0.3881×AOU | 1859 | 4.92 | 3.13 |
| 8 | 200 | 140˚E – 143˚E | DIC = 78.71 – 4.620×T + 61.42×S + 0.3851×AOU | 1859 | 4.90 | 3.30 |
| 9 | 200 | 143˚E – 144˚E | DIC = 82.38 – 4.503×T + 61.32×S + 0.3817×AOU | 1859 | 4.97 | 2.99 |
| 10 | 259 | 144˚E – 168˚E | DIC = 52.92 – 4.650×T + 62.18×S + 0.3818×AOU | 1800 | 4.95 | 2.77 |
| **Average** |  |  |  |  | 4.83 | 3.55 |

**^a^** Number of data in each testing set

**^b^** Number of data in each training set

| **Table S8.** Comparison of the rate of glacier-derived freshening (R_g_) and overall freshening (R_all_) in the SOc during 1960 ~ 2016. | | | | |
| --- | --- | --- | --- | --- |
| **Location** | **Sector** | **Period** | **R_g_ (**‰ year^-1^**)** | **R_all_ ^a^ (**‰ year^-1^**)** |
| **AP ^b^** | Atlantic | 1979-1996 | 0.2 | 0.24 |
| **AP** | Atlantic | 1996-2016 | -0.1 | -0.21 |
| **WS ^c^** | Atlantic | 1973-1989 | 0.03 | 0.09 |
| **150˚E** | Indian | 2002-2013 | 0.11 | 0.3 |
| **90˚E** | Indian | 1978-1996 | -0.03 | -0.2 |
| **CD ^d^** | Indian | 1978-2006 | 0.02 | 0.04 |
| **RS_west ^e^** | Pacific | 1996-2011 | 0.22 | 0.21 |
| **RS_east ^f^** | Pacific | 1994-2007 | 0.09 | 0.07 |
| **ABS** | Pacific | 1960-2000 | 0 | -0.03 |

**^a^** R_all_ is calculated from salinity trend based on an average salinity of 34.3 in the SOc (Eq. S12)

**^b^** Antarctic Peninsula

**^c^** Weddell Sea

**^d^** Ross Sea west

**^f^** Ross Sea east

References

1 Sarmiento, J. L. & Gruber, N. *Ocean Biogeochemical Dynamics*. (Princeton University Press, 2006).

2 Gruber, N. *et al.* The oceanic sink for anthropogenic CO_2_ from 1994 to 2007. *Science* **363**, 1193-1199, doi:10.1126/science.aau5153 (2019).

3 Lewis, E. R. W., D. W. R. Program Developed for CO_2_ System Calculations. United States. doi:10.15485/1464255 (1998).

4 Olsen, A. *et al.* GLODAPv2.2019 – an update of GLODAPv2. *Earth Syst. Sci. Data* **11**, 1437-1461, doi:10.5194/essd-11-1437-2019 (2019).

5 Olbers, D. G., Viktor V; Seiß, Guntram; Schröter, Jens. Hydrographic Atlas of the Southern Ocean in original file formats. *PANGAEA*, doi:10.1594/PANGAEA.750658 (2010).

6 Pan, X. L., Li, B. F. & Watanabe, Y. W. The Southern Ocean with the largest uptake of anthropogenic nitrogen into the ocean interior. *Sci. Rep.* **10**, 8838, doi:10.1038/s41598-020-65661-2 (2020).

7 Schlitzer, R. *Ocean Data View*, <<https://odv.awi.de>, 2020> (2020).

8 Hastie, T., Tibshirani, R. & Friedman, J. H. *The Elements of Statistical Learning: Data Mining, Inference, and Prediction*. (Springer, 2009).

9 Fushiki, T. Estimation of prediction error by using K-fold cross-validation. *Stat. Comput.* **21**, 137-146, doi:10.1007/s11222-009-9153-8 (2009).

Figures

Fig. S1


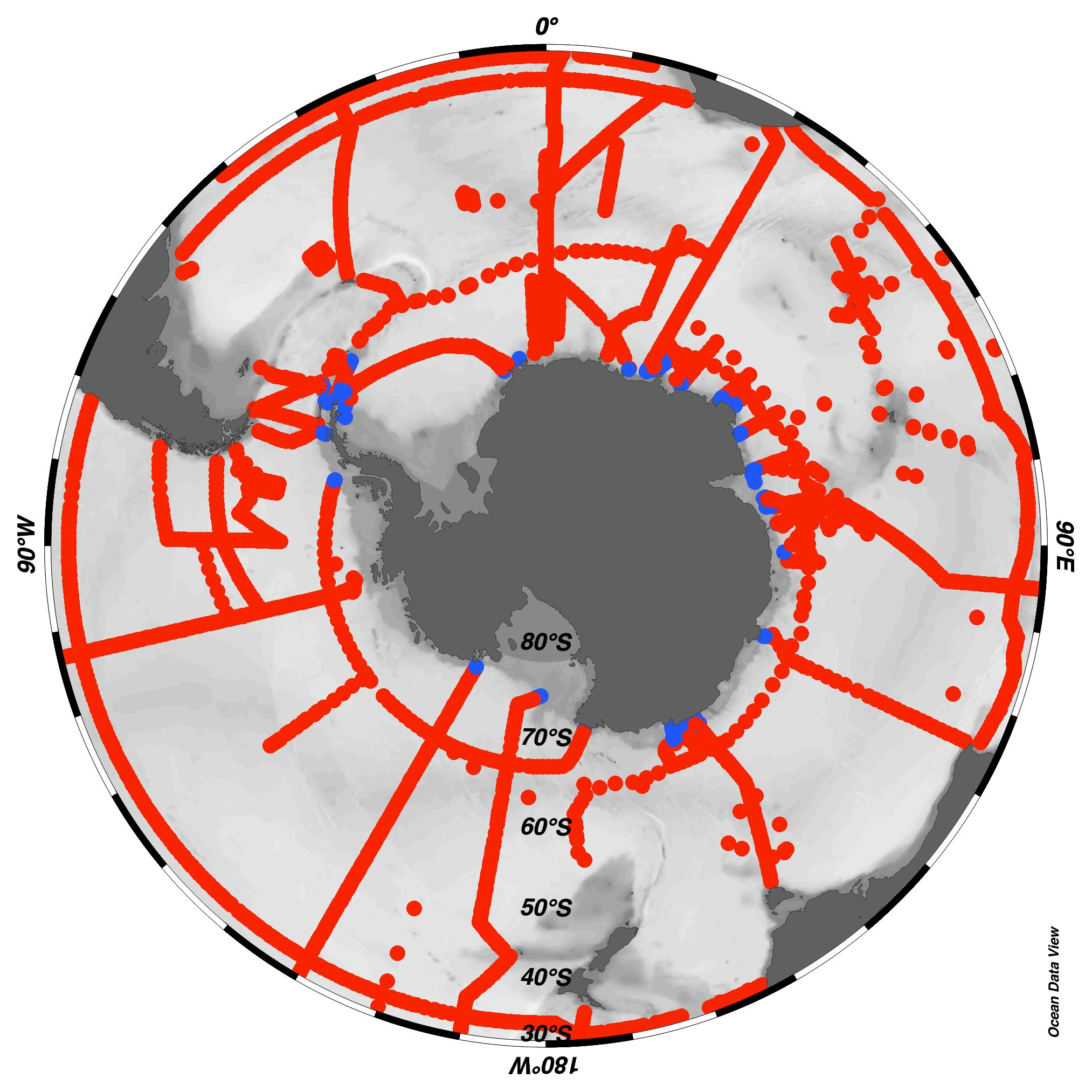


**Fig. S1. Map of cruise data used for constructing DIC parameterizations.** The data for DIC_open_ and DIC_coastal_ are shown by red and blue points, respectively. Data were obtained for the period of 2000 to 2017. See Table S1 for detailed information of these cruise data. This figure was drawn using *Ocean Data View 5.3.0 (https://odv.awi.de)* ^7^.

Fig. S2

**Fig. S2. Map of cruise data used for estimating the time-series of freshening in the SOc.** Colour bar indicates the date of each cruise. See Table S2 for detailed information of these cruise data. This figure was drawn using *Ocean Data View 5.3.0 (https://odv.awi.de)* ^7^.

Fig. S3

**Fig. S3. Relationships between DIC_pre_ and DIC_obs_ in the open SO and the SOc**. n indicates the number of data points; R^2^ indicates the coefficient of determination; and RMSE indicates the root-mean-square error.

Fig. S4

**Fig. S4. Independent validation for open SO parameterization.** Differences between DIC_obs_ and DIC_pre_ (DIC_obs_ – DIC_pre_) are calculated by using data from three independent cruises: 06AQ20071128 (Atlantic sector); 09AR20160111 (Indian sector); 49NZ20170208 (Pacific sector). Grey shadings indicate the range of ± 1 RMSE. Grey dashed lines indicate the range of ± 3 RMSE. The map on the right side shows the location of the three cruises. Map was drawn using *Ocean Data View 5.3.0 (https://odv.awi.de)* ^7^.

Fig. S5

**Fig. S5. *k*-fold cross-validation for SOc parameterization.** We divided the observational data set in the SOc into 10 testing sets and conducted cross-validations 10 times ^8,9^. Grey shadings indicate the range of ± 1 RMSE. Grey dashed lines indicate the range of ± 3 RMSE. The map in the right side shows the location of the testing sets with colour scale. Map was drawn using *Ocean Data View 5.3.0 (https://odv.awi.de)* ^7^.

Fig. S6

**Fig. S6. Self-validation of DIC_open_.** Colours of data points indicate the differences between the observed DIC (DIC_obs_) and the predicted DIC in the open SO (DIC_open_). We showed these difference in several depths: 50, 100, 200, 500, 1000, 1500, 2000, and 3000 m. These figures were drawn using *Ocean Data View 5.3.0 (https://odv.awi.de)* ^7^.

Fig. S7

**Fig. S7. Self-validation of DIC_coastal_.** Colours of data points indicate the differences between the observed DIC (DIC_obs_) and predicted DIC in the SOc (DIC_coastal_). We showed these difference in several depths: 50, 100, 200, 500, and 1000 m. These figures were drawn using *Ocean Data View 5.3.0 (https://odv.awi.de)* ^7^.

Fig. S8

**Fig. S8. Correlation of melt freshwater fraction in the SOc with SAM index.** The dashed line indicates the regression line (c.f. Fig. 2d).

Fig. S9

**Fig. S9. Four north-south vertical sections of the freshwater fraction (F_g_) estimated by this study around SOc.** The positions of these sections are shown by orange lines in the lower left map. Data are used during the whole estimation period in this study (1926–2016). Black dots indicate data points. Bold solid lines indicate the contour of F_g_ equals to zero. Gray shadings indicate no observational data. Left side of each section is the Antarctic ice sheet. Right side of each section is the boundary of SOc, which indicates the location closest to the 1,500 m bottom depth where has observational data. F_g_ at the surface gradually decreases with increasing distance from the Antarctic ice sheet and decreases to nearly zero before reaching the boundary of SOc. For easy viewing, the bathymetries are not shown in these sections. These figures were drawn using *Ocean Data View 5.3.0 (https://odv.awi.de)* ^7^.

Fig. S10

**Fig.10. Gridded data used to calculate the average F_g_ in each sector of the SOc. (a)** Distributions of the gridded data during each period, which are interpolated from the raw data shown in Fig. 2a. **(b)** Map of the 1˚ x 1˚ grid used for interpolation. **(c)** Schematic of the interpolation algorithm used in this study. Here, we used the “weighted-average gridding” method given by Ocean Data View to perform the interpolation. Blue circle indicates the grid point and red circles indicate raw data. All raw data within a given range (gray dash line box) around one grid point were used to calculate the value on this grid point. The range here was set to be 5 degrees of longitude and 1 degree of latitude. See the user guide of Ocean Data View for detail. These figures were partly drawn using *Ocean Data View 5.3.0 (https://odv.awi.de*) ^7^.
